# Polyethylene Mulch Emissions Differentially Impact the Soil Metabolome and Microbial Community in Field Pea (*Pisum sativum* L.) Cultivation

**DOI:** 10.3390/jox16020049

**Published:** 2026-03-15

**Authors:** Emoke Dalma Kovacs, Nguyen Khoi Nghia, Melinda Haydee Kovacs

**Affiliations:** 1 Research Institute for Analytical Instrumentation, National Institute for Research and Development in Optoelectronics INOE 2000, Donath 67, 400293 Cluj-Napoca, Romania; dalmaemokekovacs@gmail.com; 2 Faculty of Soil Science, College of Agriculture, Can Tho University, Campus II, 3/2 Street, Ninh Kieu District, Can Tho City 900100, Vietnam

**Keywords:** polyethylene mulch, soil metabolomics, microbial community structure, plastic-derived compounds, agricultural soil health

## Abstract

Background and Objectives: Polyethylene (PE) mulching enhances crop productivity through microclimate optimization but introduces synthetic polymer-derived compounds into agricultural soils. Despite widespread use, biochemical and microbial impacts of PE mulch emissions remain poorly understood. This study investigated the impact of PE mulch emissions on soil metabolomes and microbial communities during field pea (*Pisum sativum* L.) cultivation. Methods: A 75-day field experiment compared PE-mulched and non-mulched soils across five temporal sampling points (T0–T4). Headspace solid-phase microextraction coupled with gas chromatography–mass spectrometry was used to identify PE-derived organic compounds in mulched soils. Microbial community structure was assessed through the phospholipids derived fatty acids (PLFA) approach, whereas mass spectrometric untargeted metabolomics was used to characterize the soil biochemical profiles. Results: Analysis identified 18 PE-derived organic compounds (n-alkanes, phthalates, and additives) in the mulched soils. PE mulching significantly increased bacterial abundance (anaerobic bacteria, actinomycetes, and aerobic bacteria) but suppressed all functional fungal guilds, particularly saprotrophic fungi (30% reduction) and arbuscular mycorrhizal symbionts. PE-derived organic compounds were associated primarily with the first RDA axis (RDA1), which alone explained 44.6% of the metabolome variance. These compounds presented strong positive correlations with organic nitrogen compounds and lipids and negative correlations with benzenoids and nucleotides. Pathway analysis revealed perturbations in energy metabolism, lipid metabolism, and xenobiotic degradation pathways. Conclusions: PE mulch emissions differentially shift soil microbial communities and metabolic networks, with bacterial proliferation contrasting with fungal suppression. These findings highlight the complex trade-offs between agronomic benefits and soil biological impacts, emphasizing the need for sustainable mulching alternatives.

## 1. Introduction

Mulching is widely used to optimize soil conditions and increase crop productivity, with growing adoption as a climate-resilient strategy to buffer temperature extremes and irregular rainfall [1]. Polyethylene (PE) films dominate modern agriculture, with 2024 estimates projecting the global market to reach $12.8 billion by 2031 [2]. Low-density polyethylene (LDPE) and linear low-density polyethylene (LLDPE) provide cost-effective and durable solutions for soil temperature regulation, moisture retention, and weed suppression, contributing to substantial yield improvement [3,4]. However, PE mulch application often results in synthetic polymer loading into agricultural soils. Photodegradation by solar UV radiation, combined with thermooxidative processes, mechanical stress, and subsequent biotic degradation, causes progressive film deterioration and fragmentation. This leads to residual plastic accumulation, microplastic formation, and the release of polymer additives and degradation byproducts into the soil environment [5].

PE mulch introduction into agricultural systems influences soil physicochemical properties and biological processes. PE films modify soil microclimate conditions, altering temperature regimes and moisture dynamics while reducing evapotranspiration and gas exchange at the soil–atmosphere interface [4,6]. Beyond physical effects, PE mulch materials contain base polymers and various additives, including plasticizers, stabilizers, and processing aids [7]. These PE-derived organic compounds can leach into soil, potentially affecting ecosystem functioning and microbial diversity through xenobiotic stress responses and altered carbon substrate availability [8,9]. The soil microbiome, comprising diverse bacterial, fungal, and archaeal communities, is a critical component for nutrient transformation, organic matter decomposition, and overall ecosystem functioning [10,11]. Huang et al. [12] reported that mulching typically promotes microbial abundance, primarily through enhanced moisture retention and thermal buffering. However, PE mulch adds complexity by releasing PE-derived organic compounds and leachates [3] that may selectively pressure microbial communities through altered substrate use and stress responses [13]. Xu et al. [14] revealed that mulch film residues altered bacterial community structure and network topology, decreasing the stability of symbiotic networks.

While the net effect of mulching on total microbial biomass is often positive, the taxonomic and functional restructuring of microbial communities remains poorly characterized. Wu et al. [15] reported the highest bacterial and actinomycete abundances under PE mulch. Gao et al. [16] observed increases in *Chloroflexi* and *Firmicutes* after mulching. Rong et al. [17] showed that *Pedomicrobium* and *Nocardia* were significantly more abundant under LDPE mulch amendment. In contrast, Song et al. [18] found that plastic mulch residue altered soil fungal community composition and diversity, reducing fungal richness and shifting the community structure. Such changes in key functional guilds involved in organic matter decomposition, nutrient cycling, and secondary metabolism likely cascade into alterations of the soil metabolome. Liu et al. [19] reported that shifts in community composition coincided with changes in microbial functions, particularly nutrient cycling. Mulch-derived micro- and nanoplastics frequently affect soil nitrogen biogeochemistry [20]. Differential microbial metabolic activity could modulate the production and turnover of primary and secondary metabolites, influencing soil biochemical processes and nutrient bioavailability. Xu et al. [15] reported that mulch film residues also disturb the metabolic spectrum of soil. Understanding these community-level dynamics and their metabolomic consequences is thus critical for evaluating the long-term effects of PE mulching on soil health and ecosystem functioning. Despite increasing recognition of PE mulch impacts [21], mechanistic relationships between PE-derived organic compounds, microbial community shifts, and metabolite dynamics remain contradictory [22]: some studies describe enhanced microbial activity and biomass due to improved soil moisture and temperature conditions [3,5], whereas others note suppression of functional guilds, particularly fungal decomposers (saprotrophic *Basidiomycetes* and *Ascomycetes*) and mycorrhizal symbionts (arbuscular mycorrhizal fungi) [23]. These discrepancies likely reflect differing microbial sensitivities to PE-derived compounds, whose chemical mediators are still poorly characterized. The mechanisms driving microbial shifts—whether from direct toxic effects, altered resource competition, or modified soil chemistry—remain unclear. The temporal dynamics of soil metabolite profiles and the potential for PE-derived compounds to influence soil chemistry through sorption–desorption processes and cometabolic transformations require investigation. Clarifying relationships among PE-derived organics, metabolite profiles, and microbial functional groups is essential for understanding the cascading effects on soil ecosystems. Integrated approaches coupling chemical characterization, metabolomic profiling, and microbial community assessments are needed to disentangle these interactions and support evidence-based management strategies that reconcile crop productivity with long-term soil health.

We hypothesized that (H1) PE mulch releases a set of organic compounds into the surrounding soil, with their concentrations following time-dependent leaching and degradation dynamics that peak during active crop growth and decline following mulch removal; (H2) these PE-derived compounds, acting as xenobiotic carbon substrates, selectively shift soil microbial community composition by suppressing sensitive functional groups while enriching degrader-associated ones; and (H3) the resulting microbial shifts are reflected in measurable alterations to the soil metabolome. We therefore aimed to quantify these PE-derived compounds in soil, assess their effects on soil microbial community composition and metabolite profiles, and identify associated metabolic pathways indicative of altered soil biogeochemical functioning.

## 2. Materials and Methods

### 2.1. Soil Sampling

The leguminous plant *Pisum sativum* L. (garden pea, family Fabaceae) was grown in an open agricultural field at 45°55′06″ N, 21°21′07″ E. The experiment was conducted in 2024 over a 75-day period encompassing the entire life cycle of the pea, from presowing (before mulch application) through the postharvest period (after mulch removal). The field soil was a Pellic-Gleyic Vertisol with a pH of 6.19 (±0.42) and an organic matter content of 1.17 (±0.25)%. The experiment was laid out in a split-plot randomized complete block design with two replicate blocks. The main-plot factor was Treatment, comprising two levels: PE (polyethylene film mulching) and C (control, without mulch). Each main plot was divided into five subplots, each measuring 3 × 5 m (15 m^2^). Within each subplot, six rows were established (excluding marginal rows) with an interrow spacing of 0.5 m and an intrarow spacing of 0.25 m, resulting in 20 plants per row and 120 plants per subplot. The blocks were separated by 2 m buffer zones, and between subplots, a minimum distance of 1.5 m was considered. Prior to seeding and mulch application, chemical fertilizer was applied at recommended rates of 30, 50, and 55 kg·h^−1^ N (${NH}_{4}^{+}$), P ($P_{2}O_{5}$) and K ($K_{2}O$), respectively. The experiment was run in parallel. Bulk soil samples (soil between rows) were collected during five time periods as follows: T0—day 0 (before mulch application/before sowing); T1—day 15 (after mulching/germination); T2—day 30 (after mulching/vegetative growth); T3—day 60 (after mulching/flowering-fruiting); and T4—day 75 (after 5 days of mulch removal/harvest). Specifically, five sampling points were selected using a systematic X-shaped transect pattern to ensure spatial representativeness across the subplot areas. Soil cores from the five points were homogenized into a single composite sample per plot. This procedure was independently replicated three times per subplot.

### 2.2. Chromatographic Analysis of Polyethylene Mulch-Derived Organic Compounds

Prior to use, polyethylene mulch material was tested for potential organic compound release as described by Lattuati-Derieux et al. [24]. Briefly, 3 g of chopped mulch film was placed in 20 mL headspace vials and sealed with an aluminum crimp cap with a PTFE/silicone/PTFE septum. For extraction, a 50/30 µm divinylbenzene-carboxen/polydimethylsiloxane (DVB-CAR/PDMS, Supelco, Bellefonte, PA, USA) SPME fibre was used. Before the start of the analysis, the fibre was conditioned in the GC injection port at 270 °C according to the manufacturer’s recommendation. The vial with the sample and the inserted SPME fibre were placed in the heating block of a Triplus RSH autosampler set at 60 °C for 30 min. After this, the compounds were thermally desorbed at 270 °C for 10 min in the GC injector. To avoid carry-over effects, the fibre was baked for 5 min after the desorption cycle. The separation and quantification of the compounds were performed with a GC–MS/MS system of Ultra Trace GC 1310 gas chromatograph and a TSQ 9000 triple quadrupole mass spectrometer (Thermo Fisher Scientific, Waltham, MA, USA). The separation of the compounds was performed with a TR-1MS fused-silica capillary column of 30 m length × 0.25 mm I.D. × 0.25 µm film thickness (Thermo Fisher Scientific, Waltham, MA, USA). Helium was used as the carrier gas in constant mode with a flow rate of 1 mL·min^−1^. The injector was set at 270 °C in splitless mode. The column oven temperature programme started at 40 °C and was maintained isothermally for 10 min, followed by a gradient increase of 5 °C·min^−1^ until 270 °C. This final temperature was maintained isothermally for another 10 min. The transfer line was set at 270 °C, and the ion source was set at 280 °C. The mass spectra were acquired under electron ionization mode (EI) at 70 eV and recorded in the range of 50–500 *m*/*z* at one cycle per second. The same analytical procedure was used for the soil samples. The analytical procedure was validated by artificially spiking diatomaceous soil with the identified representative mulch material, which emitted organic compounds such as n-alkanes, phthalates, and volatile compounds.

### 2.3. Soil Microbiome Phenotypic Structure Assessment

Microbiome phenotypic structure assessment was performed by phospholipid fatty acid (PLFA) profiling approach according to the method described by Kovacs et al. [25]. Briefly, lipids were extracted with modified Blight and Dyer solvents and fractionated on a silica column into glycolipids, phospholipids, and neutral lipids. Phospholipids were converted into fatty acid methyl esters (FAME) through trans-esterification and analyzed by gas chromatography–flame ionization detection (GC–FID). Specific PLFA signatures were classified into bacterial and fungal functional groups by the MIDI Sherlock™ Microbial Identification System (Microbial ID, Newark, DE, USA). The abundance of each microbial guild was calculated and reported as nmol·g^−1^ dry soil.

### 2.4. Mass Spectrometric Identification of Soil Untargeted Metabolite Profiles

Soil untargeted metabolite profiles were analyzed after extraction from 1 g of lyophilized soil samples as previously described by Kovacs et al. [26]. Briefly, the extracts were split for complementary mass spectrometric analysis. In the case of GC–MS/MS, the aliquots underwent two-step derivatization with methoxyamine hydrochloride followed by MSTFA. The analysis employed a Thermo Trace 1310 GC coupled to a TSQ 9000 triple quadrupole MS (Trace 1310 GC-TSQ 9000 MS, Thermo Scientific, Waltham, MA, USA) with an HP-5MS capillary column (30 m × 0.25 mm i.d., 0.25 µm film thickness). The instrument operation conditions were the same as those previously reported [26]. Mass spectra were acquired in electron ionization mode (70 eV, ion source 230 °C, *m*/*z* 50–550, solvent delay 3.5 min). Data processing was performed with Xcalibur 4.0 and MS-DIAL version 4.9 as previously described [27]. For MALDI-TOF/TOF MS, the dried extracts were reconstituted in TFA/water/ammonium hydroxide (0.05%/97.95%/2%, *v*/*v*/*v*; 25 µL) and mixed 1:1 with a 9-aminoacridine matrix (10 mg/mL^−1^ in 0.1% TFA/acetone). One microliter of each sample mixture was spotted onto polished steel targets (MTP 384, Bruker, Bremen, Germany) and analyzed with an Autoflex maX MALDI-TOF/TOF (Bruker Daltonics, Bremen, Germany) with a Smartbeam-II Nd:YAG laser (355 nm) in linear negative ion mode following external calibration with a standard mixture of metabolites (lactate, succinate, malate, AMP, ATP, and CoA). The acquisition parameters included 35% laser intensity, 500 Hz frequency, and ~2000 shots per spectrum. Quality control assurance was performed by external calibration verification before each analytical batch to ensure mass accuracy and by matrix-only controls to identify background signals and potential contaminants. Spectral processing was performed with flexAnalysis 3.4 (centroid detection with TopHat baseline correction), whereas metabolite annotation was performed using the R-MetaboList 2 and rMSIfragment tools as described by Lai et al. [27], Baquer et al. [28], and Peris-Diaz et al. [29].

### 2.5. Statistical Analysis

Statistical analysis was conducted with R software (v.4.3.3, R Core Team). Prior to inferential testing, the assumptions for normality and homogeneity of variance were verified for each response variable using the Shapiro–Wilk with the Shapiro.test() function, and Levene’s tests with leveneTest() function, (α = 0.05), respectively. Two-way analysis of variance (ANOVA) was performed with the aov() function from base R to evaluate the main effects of Group (*G*: PE mulch vs. Control), Time (*T*: T0-T4), and their interaction (*G* × *T*) on the polyethylene mulch-derived organic compounds and microbiome phenotypic structure abundances in the soil. Following significant ANOVA results, Tukey’s HSD post hoc tests were conducted with the Tukey HSD() function to perform pairwise comparisons between the two groups at each time point. Statistical significance was set at α = 0.05, with significance levels denoted as * *p* < 0.05, ** *p* < 0.01, and *** *p* < 0.001. The metabolomic data for the heatmap were pre-processed with log-transformation to reduce heteroscedasticity and right-skewed distributions, followed by z-score normalization ($z=\frac{x-\mu }{\sigma })$ across samples using the scale() function to standardize metabolite variance across samples and allow cross-comparisons. Unsupervised hierarchical clustering of metabolites was performed using the Euclidean distance as the dissimilarity metric and the complete linkage method (hclust function) to identify metabolites with similar abundance patterns across PE and C soils. Metabolite clustering quality was assessed by calculating within-cluster Pearson correlation coefficients. Heatmap visualization was implemented in R with the pheatmap package with colour palettes from RColorBrewer (diverging the blue–white–red scheme from the RdBu palette). Heatmaps display metabolites in rows (clustered) and samples in columns (time-line order), with annotations indicating groups (PE vs. C) and metabolite superclasses. Principal component analysis (PCA) was performed on the metabolites. The data were mean-centred and scaled to unit variance prior to analysis of the prcomp function with singular value decomposition. Sample distributions were visualized with ggplot2 with a 68% confidence ellipse (normal distribution corresponding to ±1 SD, reflecting within-group dispersion) overlaid for each time point to assess temporal clustering patterns. Permutational multivariate analysis of variance (PERMANOVA) was conducted with adonis2 on Euclidean distances with 999 permutations to test group separation, temporal effects, and their interactions (*p* < 0.05). The homogeneity of multivariate dispersion was verified using betadisper() and permutest() to ensure the significant PERMANOVA results reflected true centroid differences rather than dispersion artefacts. Redundancy analysis (RDA) was performed with the vegan package to quantify the proportion of variance in microbiota and metabolite composition explained by PE-derived organic compounds. The statistical significance of the RDA models was evaluated through permutation tests (999 permutations) with the anova.cca() function. Adjusted R^2^ values were calculated with RsquareAdj() to account for the number of explanatory variables. Correlation matrices were visualized as heatmaps with ggplot2 and reshape packages, with colour gradients representing correlation strength and direction. Significance was determined at α = 0.05 and *p*-values adjusted for multiple comparisons using the Benjamini–Hochberg false discovery rate (FDR) correction.

## 3. Results

### 3.1. Organic Compounds Emitted from Polyethylene Mulch in Soil

**Figure 1:**
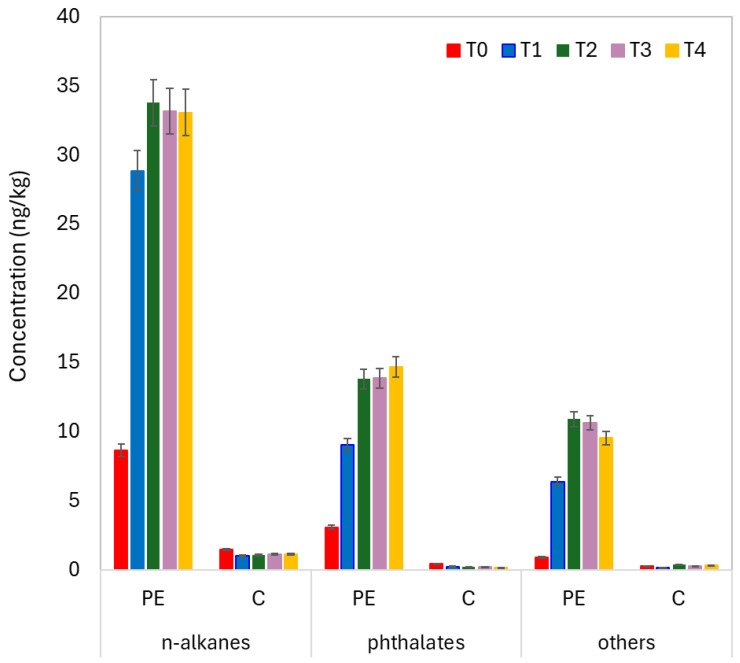
Accumulation patterns of the mean summed concentrations of polyethylene mulch-derived organic compound classes in mulched (PE) and nonmulched soils.

Analysis of field-applied polyethylene mulch revealed 18 organic compounds, comprising nine n-alkanes (n-undecane, n-dodecane, 2-methyl-dodecane, n-heptyl cyclohexane, n-tridecane, n-tetradecane, n-pentadecane, n-hexadecane, n-nonyl cyclohexane), six phthalates (dimethyl phthalate, di-n-octyl phthalate, diethyl phthalate, dibutyl phthalate, benzyl butyl phthalate, bis-2-ethylhexyl-phthalate), and three compounds from other chemical classes (acetyl tributyl citrate, bis-2-ethylhexyl adipate, naphthalene). These polyethylene mulch-associated organic compounds were detected in field soil samples throughout the pea (*Pisum sativum* L.) cultivation period. The n-alkanes were most abundant, with summed concentrations averaging 1–33.8 ng·kg^−1^, followed by phthalates, with summed average concentrations ranging from 0.2 to 14.7 ng·kg^−1^ (Figure 1).

Two-way ANOVA (Table 1) revealed significant group effects (G: mulched soil (PE) vs. control (C) nonmulched soil) for all 18 compounds (*p* < 0.001 for 16 compounds, *p* < 0.01 for 2-methyl-dodecane and n-hexadecane). The effect size ($\eta^{2}$) for group (G) was very large (0.718). Significant mulching time effects (T) were observed for 13 compounds, with n-dodecane, n-heptyl-cyclohexane, and n-pentadecane exhibiting the strongest time-dependent variation (*F*_(4,40)_ > 15, *p* < 0.01, $\eta^{2}$ = 0.427). The group × time interactions (G × T) were highly significant (*p* < 0.001, $\eta^{2}$ = 0.418) for alkanes, cycloalkanes, and most phthalates, indicating differential temporal accumulation patterns between the groups. Tukey’s HSD post hoc comparisons demonstrated that the baseline differences (T0, premulching) were generally nonsignificant or weakly significant, whereas the concentrations in the mulched soil samples (PE) were significantly greater than those in the controls (C) from T1 onwards for the majority of the compounds.

### 3.2. Soil Microbiome Phenotypic Structure Abundance

PE had divergent effects on the soil microbial communities, significantly increasing bacterial abundance while suppressing fungal populations (Table 2). Two-way ANOVA revealed significant effects of mulch-derived organic compounds on the abundance of all microbial groups (*F*_(1,40)_ = 4.2–143.9, *p* < 0.05). The bacterial communities substantially increased under polyethylene mulching. Anaerobic bacteria presented the strongest response (*F*_(1,40)_ = 143.9, *p* < 0.001), with the abundance in the mulched plots being 13.6 units greater than that in the control plots. Similarly, actinomycetes and aerobic bacteria were significantly elevated in the mulched soil (Δ = 12.8 units and Δ = 12.4 units, respectively). Conversely, all fungal groups presented significantly lower abundances in the mulched soil than in the control soil. Saprotrophic fungi showed the strongest suppression (*F*_(1,40)_ = 143.9, *p* < 0.001, Δ = 1.96 units), followed by arbuscular mycorrhizal fungi and ectomycorrhizal fungi. Significant Group × Time interactions were detected for aerobic bacteria (F_4,40_ = 5.79, *p* < 0.001), anaerobic bacteria (*F*_(1,40)_ = 9.4, *p* < 0.001), and actinomycetes (*F*_(1,40)_ = 5.43, *p* < 0.001), indicating temporal variation in treatment response.

### 3.3. Soil Metabolite Characteristics

**Figure 2:**
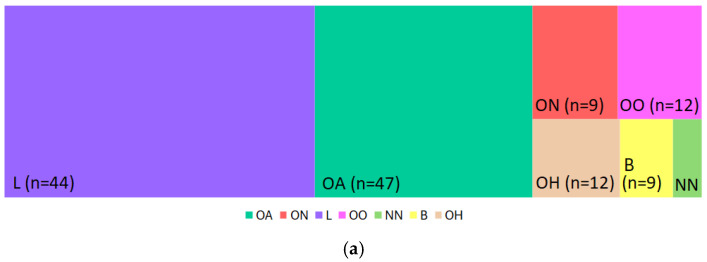

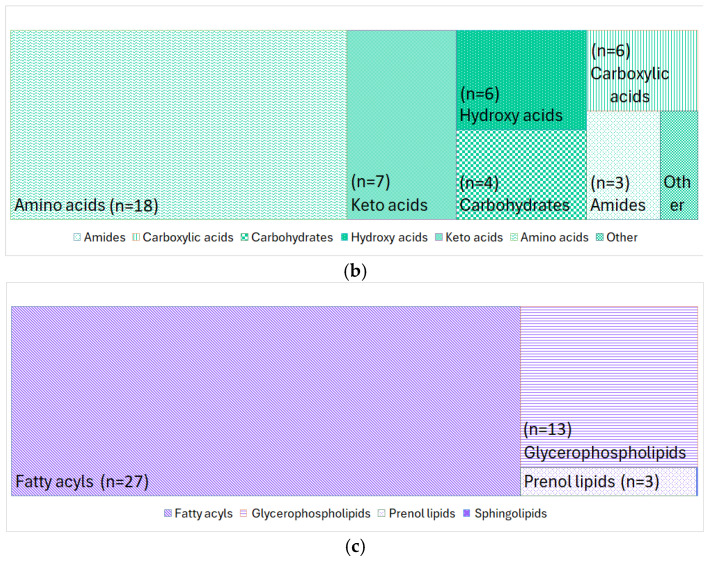
Tree map distribution of soil metabolites: (**a**) all identified superclasses; (**b**) chemical classes of metabolites that belong to the OA superclass; (**c**) chemical classes of metabolites that belong to the L superclass. Metabolite superclass abbreviations: OA—organic acids and derivatives; ON—organic nitrogen compounds; L—lipids and lipid-like molecules; OO—organic oxygen compounds; NN—nucleosides, nucleotides, and analogues; B—benzenoids; and OH—organoheterocyclic compounds.

Mass spectrometric analysis identified 241 metabolites in the soil samples, with 133 of these metabolites having KEGG annotations; therefore, the subsequent analysis was restricted to these annotated metabolites. The identified metabolites were classified into seven subclasses according to established chemical ontology frameworks: organic acids and derivatives (OAs); organic nitrogen compounds (ONs); lipids and lipid-like molecules (L); organic oxygen compounds (OOs); nucleosides, nucleotides, and analogues (NNs); benzenoids (B); and organoheterocyclic compounds (OHs). The percentage distribution of soil metabolites across these superclasses is presented in Figure 2a. The L class constituted the largest fraction, accounting for 44.5% of the total metabolite abundance, followed by OA at 31.2%. The detected OAs (*n* = 47) were partitioned among seven classes (Figure 2b) in the following pattern: amino acids (49%) > keto acids (15.8%) > hydroxy acids (10%) > carbohydrates (9%) > carboxylic acids (6.9%) > amides (6.1%) > other organic acids (3.1%). L (*n* = 44) superclass metabolites were grouped into four classes (Figure 2c), of which fatty compounds were the most prevalent, followed by glycerophospholipids. The metabolomic profile also revealed diverse chemical compositions through the OO (25.74%, *n* = 12), OH (18.59%, *n* = 12), and ON (26.39%, *n* = 5) superclasses. Within the OH superfamily, pyrimidines presented the highest relative abundance (29.85%), followed by lactones (25.66%) and pyridines (12%). The ON superclass compound fraction was predominantly composed of quaternary ammonium salts (57.41%). Additional minor constituents included compounds of the B (11.21%) and NN (6.14%) superclasses.

### 3.4. Differentially Expressed Metabolites

**Figure 3:**
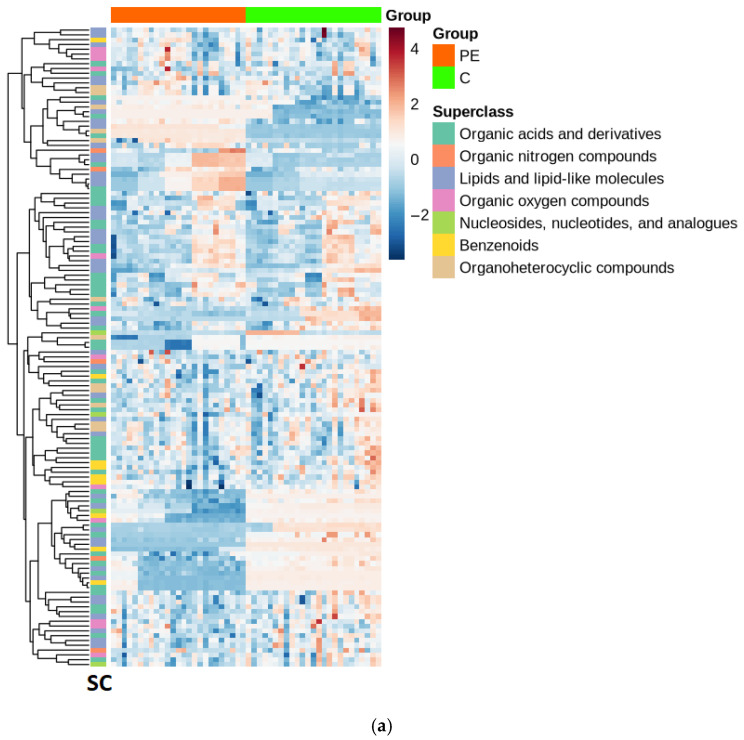

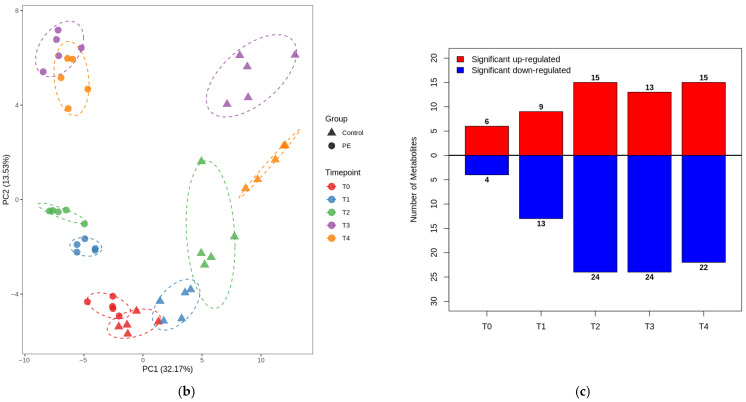
Soil metabolite variation in PE and C soils. (**a**) Hierarchical clustering heatmap analysis of differential metabolite abundance between PE and C soils with metabolite superclass annotation. (**b**) Principal component analysis of temporal metabolomic variation in PE and C soils along the established T0–T4 timeline. (**c**) Temporal dynamics of significantly upregulated and downregulated metabolites in PE soils relative to those in C soils across T0–T4.

The heatmap (Figure 3a) presents a comparative metabolomic analysis of polyethylene mulched soil (PE) and control (C) nonmulched soil across a temporal gradient. The colour gradient represents the relative metabolite abundance, with red indicating elevated levels and blue indicating reduced levels relative to the mean. The sample columns were ordered chronologically without clustering to preserve temporal relationships. Unsupervised hierarchical clustering with Euclidean distance metrics and complete linkage agglomeration identified metabolite coexpression patterns and functional relationships. The analysis revealed treatment-dependent metabolic restructuring. The dendrogram on the left side illustrates the hierarchical relationship among metabolites with branch heights ranging from 0.21 to 14.0 (Euclidean distance units), representing the dissimilarity between metabolite clusters. At a broad level (k = 3), the dendrogram resolves into three major clusters containing 88, 27, and 18 metabolites, whereas finer resolution (k = 5) reveals five distinct clusters (38, 50, 12, 18, and 15 metabolites) with varying degrees of internal coherence: the mean within-cluster Pearson correlations range from 0.217 to 0.709, with the most tightly coregulated cluster (*n* = 12) showing a mean correlation of 0.709 (median = 0.627, range: 0.436–1.0), indicating strongly coordinated abundance patterns. These clusters represent groups of metabolites exhibiting similar abundance patterns across samples. A substantial cluster of OAs decreased throughout the PE soil group. Conversely, certain ON and OH metabolites appear relatively elevated or unchanged in PE soils. C group soils display more heterogeneous patterns, with both increases and decreases across metabolite classes.

The PCA biplot demonstrates temporal metabolomic divergence between PE and C soils across the five time-line points (Figure 3b). The first two principal components (PC1: 32.17%, and PC2: 13.53%) collectively explained 45.7% of the total variance. Figure 3b reveals three distinct phases: first, an initial convergence at T0 and T1, where both treatments cluster together in the lower-central region, indicating metabolomic similarity before and shortly after mulch application; second, a progressive divergence through T2 and T3, with maximal separation occurring at T3, where PE samples occupy the upper-left quadrant while controls shift to the upper-right quadrant along PC1; and third, a persistent differentiation at T4, where PE samples form a tight diagonal cluster despite mulch removal, suggesting legacy effects on soil metabolomes. PC1 primarily captures temporal progression, with early timepoints at negative values and later stages at positive values. PC2 discriminates against treatment effects, particularly at T3, where the PE and C groups occupy opposite quadrants. The 95% confidence ellipses show within-group homogeneity and clear between-group separation, with group-specific metabolomic trajectories becoming increasingly distinct over time. The bar chart of the upregulated metabolites (Figure 3c) reveals progressive metabolomic differentiation from T0 to T4, with the downregulated:upregulated ratio increasing from 0.67 at T0 to a peak at 1.85 at T3. Maximum metabolomic suppression occurred at T2 and T3 (24 downregulated metabolites). The minimal differential regulation at T0 (six up, four down) confirms baseline metabolomic similarities before polyethylene mulch application.

### 3.5. Relationships Among Soil Physicochemical Properties, Metabolites, and Microbial Communities

**Figure 4:**
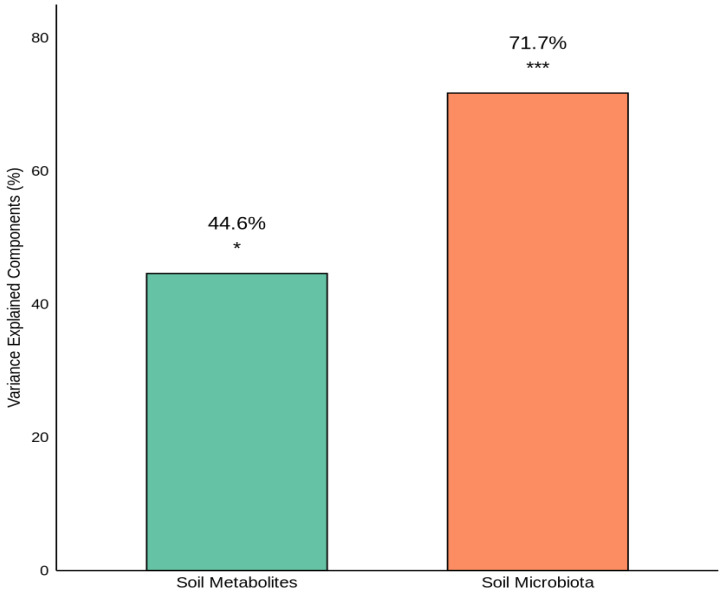
RDA variance in the soil microbiota and metabolites explained by PE-derived organic compounds (* *p* < 0.5, *** *p* < 0.001).

Redundancy analysis (RDA) was employed to quantify the proportion of variance in the soil microbiota composition and the soil metabolite profiles explained by the PE-derived organic compounds (Figure 4). The analysis revealed that these compounds accounted for 71.7% of the variance in the soil microbiota composition (*p* < 0.001), demonstrating highly significant explanatory power. In contrast, the compounds explained 44.6% of the variance in the soil metabolite profiles (*p* < 0.05), indicating a moderate but statistically significant influence. The substantially greater variance explained in the microbiota than in the metabolites suggests that mulch-derived organic compounds exert stronger direct selective pressure on the microbial community structure than on the soil metabolome.

**Figure 5:**
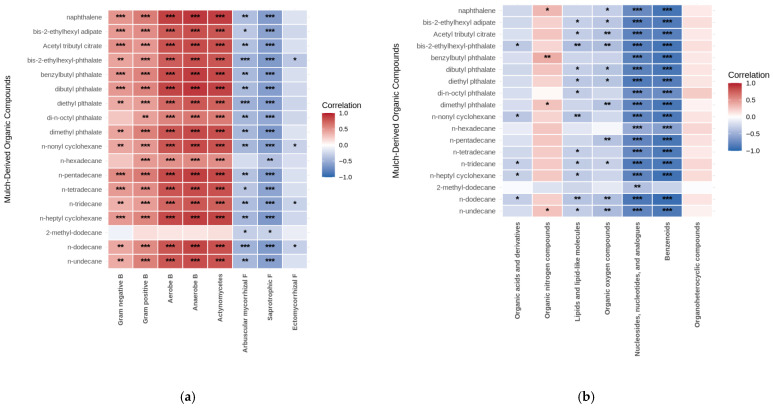
Pearson correlation matrices: (**a**) Pearson correlations between PE-derived organic compounds and soil microbial groups; (**b**) Pearson correlations between mulch-derived organic compounds and soil metabolite categories. (* *p* < 0.5, ** *p* < 0.01, *** *p* < 0.001, and ns—nonsignificant.)

Next, Pearson correlation coefficients were calculated to identify specific linear relationships between individual PE-derived organic compounds and soil microbial groups or metabolite categories, providing detailed insights into the pairwise associations underlying the overall patterns revealed by redundancy analysis (Figure 5). The Pearson correlation analysis between the 17 mulch-derived organic compounds and the bacterial and fungal phenotypes revealed distinct response patterns (Figure 5a). Phthalate esters, including dibutyl phthalate, benzylbutyl phthalate, and dimethyl phthalate, presented strong positive correlations with aerobic bacteria (r = 0.86–0.91, *p* < 0.001), anaerobic bacteria (r = 0.84–0.89, *p* < 0.001), and actinomycetes (r = 0.81–0.85, *p* < 0.001). Alkanes (n-dodecane, n-pentadecane, n-tetradecane, and n-undecane) presented similarly strong positive associations with these bacterial groups (r = 0.80–0.88). Conversely, these compounds demonstrated strong negative correlations with saprotrophic fungi (r = −0.58 to −0.65) and arbuscular mycorrhizal fungi (r = −0.40 to −0.62). Gram-positive and Gram-negative bacteria presented moderate positive correlations with most mulch compounds (r = 0.40–0.62). Naphthalene was strongly positively correlated with bacterial groups (r = 0.78–0.84) and negatively correlated with fungi (r = −0.54 to −0.58). The compound 2-methyl-dodecane displayed anomalously weak correlations across all the microbial groups (r = −0.36 to 0.38). Correlation analysis between the PE-derived compounds and the major metabolite categories revealed compound class-specific associations (Figure 5b). Benzenoids exhibited predominantly strong negative correlations with mulch compounds, ranging from r = −0.57 to −0.84, with naphthalene showing the strongest negative association (r = −0.84). Nucleosides, nucleotides, and analogues demonstrated similarly strong negative correlations across most compounds (r = −0.46 to −0.86). The organic nitrogen content was moderately to strongly positively correlated with phthalates and alkanes (r = 0.28–0.68). Lipids and lipid-like molecules exhibited moderate positive associations with most mulch compounds (r = 0.22–0.59). Organic acids and their derivatives displayed weak negative correlations with most compounds (r = −0.10 to −0.13), whereas some phthalates presented moderate positive correlations (r = 0.53). Organoheterocyclic compounds and organic oxygen compounds generally showed weak to moderate correlations (r = −0.27 to 0.48).

### 3.6. Metabolic Pathways

**Figure 6:**
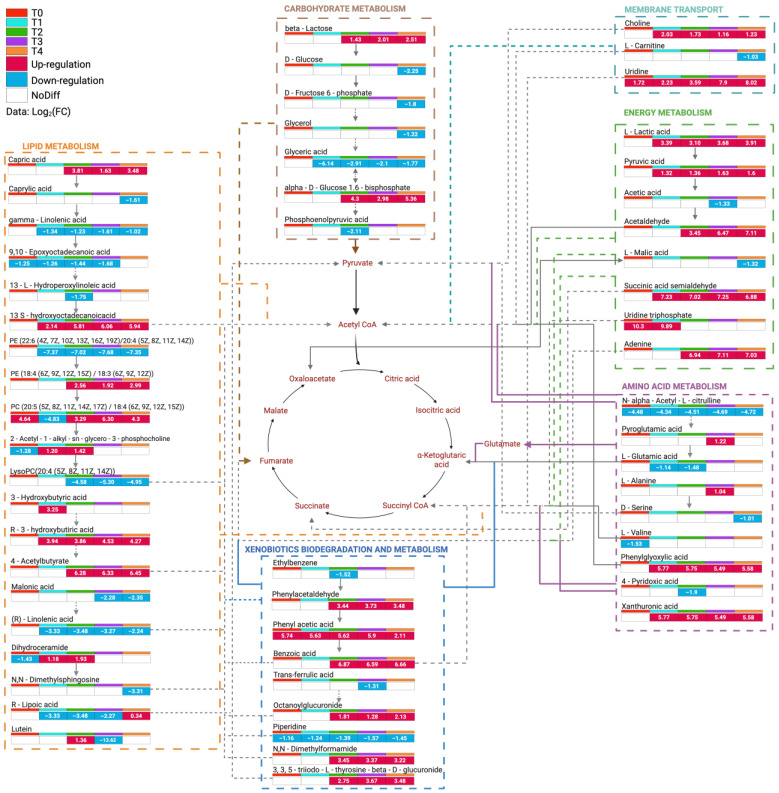
Temporal dynamics of metabolic pathway perturbations in PE-mulched vs. nonmulched soils (C) across the *Pisum sativum* L. cultivation period (T0–T4).

Pathway enrichment analysis revealed perturbations in multiple interconnected metabolic networks in response to PE mulch application (Figure 6). Energy metabolism intermediates displayed variable responses across the experimental timeline (T0–T4), with log2-fold changes ranging from moderate suppression to substantial upregulation depending on the specific metabolite and time point. Carbohydrate metabolism demonstrated treatment-dependent alterations. Lactose was consistently upregulated with increasing PE-mulching time (log_2_FC: 1.43–2.51), whereas glucose was markedly downregulated at T2 (log_2_FC: −2.28). Phosphoenolpyruvate, an important glycolytic intermediate linking carbohydrate metabolism to the TCA cycle, was significantly downregulated. Lipid metabolism presented relevant perturbations among all the metabolic pathways examined. Phosphatidylethanolamine (PE), a major structural component of cellular membranes, was strongly downregulated (approximately two times smaller). In contrast, 13-S-hydroxyoctadecanoic acid, a fatty acid oxidation product, was strongly upregulated (log_2_FC: ≈6 until the end of the experiment). Malonic acid, a competitive inhibitor of succinate dehydrogenase in the TCA cycle, was downregulated. The adaptive response to PE-mulching treatment was shown through energy metabolism. Lactic acid, an end product of anaerobic glycolysis, presented substantial upregulation (log_2_FC: ≈4 in T4). Acetaldehyde presented pronounced upregulation, with log_2_FC values varying between 3.4 and 7.1 from T2–T4. Uridine triphosphate, a critical energy and RNA precursor, was significantly upregulated at the early stages of the experiment (T0, T1). Amino acid metabolism demonstrated selective regulation patterns. N-α-Acetyl-L-citrulline was consistently downregulated across time points, with an average log_2_FC of 4.5. Glutamic acid, a central amino acid in nitrogen metabolism and the biosynthesis of biomediators, presented moderate downregulation in T1 and T2. The xenobiotic biodegradation pathway was significantly activated in PE-mulched soils. Phenylacetaldehyde, an aromatic degradation intermediate, was consistently upregulated from T2 until T4. Piperidine, an N-containing heterocyclic compound, was markedly downregulated throughout all the experiments. The integrated metabolic network analysis revealed coordinated regulation across multiple pathways, with particularly strong interconnections between central carbon metabolism (glycolysis, TCA), lipid metabolism, and xenobiotic degradation pathways.

## 4. Discussion

### 4.1. Mulching Impact on the Soil Microbiome and Soil Metabolome Profile

The present study demonstrated that PE mulch application substantially restructured soil microbial communities and metabolite profiles. PE mulch film-associated organic compounds (alkanes, phthalates, additives) detected in mulched soils confirmed continuous leaching from the polymer matrix (Figure 1), as was previously widely reported in different agricultural soils by Reay et al. [30], Scopetani et al. [31], and Viljoen et al. [32]. Compounds showed temporal accumulation from baseline (T0) onwards, indicating progressive release through photodegradation and thermo-oxidative processes [33] under field conditions [34]. According to Felgel-Farnholz et al. [35], the predominance of n-alkanes over phthalates reflects the compositional characteristics of LDPE and LLDPE formulations, where alkanes serve as processing aids and slip agents, whereas phthalates function primarily as plasticizers.

Increased bacterial populations in mulched soils—particularly anaerobic bacteria (approximately 2-fold), actinomycetes, and aerobic bacteria (Table 2)—reflect combined influences of improved microenvironmental conditions [30] and compound-specific pressures [31]. These enhancements align with documented mulching effects on soil microclimate [36], including increased soil moisture retention [37], moderate temperature fluctuations [38], and reduced evaporation [39]. Wang et al. [36] and Gao et al. [38] reported increased microbial biomass under PE mulch, attributing these changes primarily to favourable moisture and temperature regimes promoting microbial growth and metabolic activity. However, the strong correlations observed between bacterial abundance and specific PE mulch film-derived organic compounds (Figure 5a) suggest that chemical selective pressures also drive community restructuring. The capacity of certain bacterial taxa to metabolize phthalates through ester hydrolysis and subsequent aromatic ring cleavage has been documented by Boll et al. [40], potentially explaining these positive associations. Furthermore, alkanes can be degraded by terminal or subterminal oxidation pathways through alkane hydroxylase enzyme systems present in diverse bacterial lineages, including Actinobacteria [41], Proteobacteria [42], and Firmicutes [43]. Elevated actinomycete abundance is particularly noteworthy given their catabolic versatility and secondary metabolite production [44,45], facilitating adaptation to xenobiotic stress [46]. While our study quantified broad functional groups, the responses are likely species-specific or even strain-specific. High-throughput sequencing approaches have revealed that mulching can selectively enrich specific bacterial genera, such as Bacillus, Pseudomonas, and Streptomyces, while suppressing others [41,42,43].

Conversely, fungal communities presented consistent suppression across all guilds (Figure 5a). Saprotrophic fungi declined most (30% lower), followed by arbuscular mycorrhizal fungi and ectomycorrhizal fungi (Table 2), with reductions strongly negatively correlated with compounds promoting bacterial growth (Figure 5a). This differential response reflects fundamental physiological differences between bacteria and fungi. The fungi exhibit lower xenobiotic tolerance due to differences in cell wall structure, membrane composition, and detoxification enzymes [47]. Additionally, Jiang et al. [48] reported that phthalates disrupt fungal membrane integrity and interfere with the biosynthesis of ergosterol, a sterol unique to fungal membranes [49,50]. The suppression of mycorrhizal fungi is concerning agroecologically, as these symbionts facilitate plant nutrient acquisition, water uptake, and stress tolerance. However, PE mulching enhances crop productivity through improved water use efficiency, weed suppression, and soil temperature optimization [36,37,38,39]. In pea cultivation, mulching increases yields by 15–40% [41], suggesting that agronomic benefits may outweigh negative effects on specific microbial guilds, at least in the short term [51].

Metabolomic analysis revealed that PE-derived compounds explained 44.6% of the variance in the soil metabolite profiles (Figure 4), indicating a moderate but significant influence on soil chemistry. Metabolite composition (Figure 2a) was dominated by lipids and lipid-like molecules (44.5%) and organic acids and their derivatives (31.2%), reflecting their central role in microbial metabolism and soil organic matter dynamics. Hierarchical clustering of metabolites into distinct coexpression groups suggests coordinated regulation driven by shifts in microbial community composition and activity. Strong positive correlations between PE-derived compounds and organic nitrogen compounds and lipids (Figure 5b) suggest enhanced microbial biosynthetic activity [49], possibly as an adaptive response to xenobiotic stress [52] or increased microbial biomass under favourable mulching conditions [30]. Conversely, negative correlations with benzenoids and nucleosides/nucleotides indicate suppressed aromatic compound production and nucleotide metabolism, which may reflect altered microbial community function [53] or direct inhibitory effects [54,55]. Similar perturbations have been reported in soils exposed to microplastics [56] and plastic additives [49,57]. Temporal dynamics in microbial communities and metabolite profiles reveal mulching effects as dynamic processes with progressive community and metabolic restructuring throughout the growing period. These findings highlight the complex trade-offs associated with PE mulch use in agriculture, as highlighted by Sun et al. [37]. While mulching provides agronomic benefits through microclimate optimization and weed control, PE-derived compounds reshape soil microbial communities and metabolic functions. Long-term sustainability requires balancing productivity gains against potential cumulative effects on soil biological health, particularly given the observed suppression of beneficial mycorrhizal fungi. Future research should evaluate biodegradable mulch alternatives for maintaining agronomic benefits while minimizing soil biochemical impacts.

### 4.2. Potential Consequences for Soil Metabolic Pathways

Pathway enrichment analysis revealed coordinated perturbations across multiple interconnected metabolic networks (Figure 6), reflecting adaptive responses to PE mulch-derived compounds and associated shifts in microbial community composition. These metabolic alterations provide mechanistic insights into how PE mulching influences soil biogeochemical cycling and microbial energy acquisition strategies.

Carbohydrate metabolism presented treatment-dependent modulation with contrasting responses among key intermediates. Consistent lactose upregulation (log_2_FC: 1.4–2.5 from T2 to T4) suggests increased disaccharide availability, potentially derived from increased microbial exopolysaccharide production [58,59] or altered plant root exudation patterns under mulched conditions serving as a carbon source for specific microbes [60]. Conversely, the periodic glucose (in T4) and phosphoenolpyruvate (in T2) downregulation indicates decreased microbial glycolysis rate, the primary catabolic pathway (Embden–Meyerhof–Parnas pathway) for sugars breaking down into energy [61], an effect often observed when environmental factors shift the metabolic strategies [62]. According to Daunoras et al. [63], this apparent contradiction may reflect temporal metabolic switching, where glucose depletion triggers alternative carbon utilization strategies, including lactose catabolism through β-galactosidase activity. Phosphoenolpyruvate downregulation, a critical branch point linking glycolysis to the TCA cycle and serving as a phosphate donor in the phosphotransferase system, suggests reduced carbon flux toward central energy metabolism based on the findings of Gruning et al. [64]. This metabolic constraint may represent an adaptive response to xenobiotic stress, redirecting cellular resources from growth-oriented metabolism toward maintenance and detoxification processes.

The pronounced lipid metabolism perturbations represent the most significant metabolic responses observed. Phosphatidylethanolamine species, major structural components of bacterial membranes that play crucial roles in membrane curvature, protein anchoring, and stress adaptation, exhibit differential regulation [65]. The pattern suggests membrane lipid composition rather than uniform suppression. While some species were significantly downregulated, potentially reflecting the observed fungal community decline, as fungi typically maintain higher phospholipid content and contribute substantially to soil phospholipid pools [66], the upregulation of other species may indicate adaptive responses by specific microbial populations [67,68] or community composition shifts favouring organisms with distinct lipid profiles [69]. Additionally, Wang et al. [36] demonstrated that phthalate-induced membrane disruption may trigger compensatory adjustments in membrane lipid composition, with cells favouring alternative phospholipid species or adjusting fatty acid saturation profiles to maintain membrane integrity under xenobiotic stress [70]. Strong upregulation of 13-S-hydroxyoctadecanoic acid (log_2_FC: ≈6 from mid-experiment onwards) indicates increased fatty acid β-oxidation. This oxylipin, produced through lipoxygenase-mediated linoleic acid oxidation, serves as a signalling molecule in plant–microbe interactions and stress responses [71], potentially reflecting increased oxidative stress or enhanced microbial defensive response to PE-derived compounds.

Energy metabolism pathways revealed adaptive shifts toward anaerobic processes. Substantial lactic acid increase (log_2_FC: ≈4) indicates increased fermentative metabolism, consistent with observed anaerobic bacterial abundance increase. Lactic acid accumulation typically occurs under oxygen-limited conditions or when pyruvate oxidation through the TCA cycle is constrained [72]. This metabolic shift may result from PE mulch-induced alterations in soil aeration, as plastic films reduce gas exchange at the soil–atmosphere interface, potentially creating localized anaerobic microsites [4,6]. Pronounced acetaldehyde increase (log_2_FC: 3.4 from T2 to 7.1 from T4) further supports enhanced fermentative activity, as this compound serves as an intermediate in the ethanol and mixed-acid fermentation pathways [73]. However, according to Nie et al. [74], acetaldehyde can also arise from threonine catabolism and serves as a precursor for acetyl-CoA synthesis, suggesting metabolic flexibility in carbon utilization strategies. Early uridine triphosphate upregulation (T0, T1) indicates enhanced nucleotide metabolism and RNA synthesis during the initial adaptation phases [75], reflecting increased transcriptional activity associated with stress response gene expression and metabolic reprogramming [76,77].

Amino acid metabolism demonstrated selective regulation patterns indicative of nitrogen cycling perturbations. Consistent N-α-acetyl-L-citrulline downregulation (Figure 6) suggested reduced arginine metabolism through the urea cycle, important for nitrogen assimilation and polyamine biosynthesis in soil microorganisms [78]. Glutamic acid, a central nitrogen metabolism hub serving as an amino group donor and precursor for biosynthetic pathways [79], exhibited moderate downregulation during T1–T2, indicating reduced nitrogen assimilation capacity or altered partitioning in response to PE-derived compounds. Since glutamate is a precursor for γ-aminobutyric acid (GABA), glutamine, proline, and arginine biosynthesis, its downregulation could cascade through nitrogen-dependent processes, including stress tolerance and secondary metabolite production [80].

The xenobiotic biodegradation pathway provides direct evidence of microbial metabolic responses to PE-derived compounds. Phenylacetaldehyde, an aromatic degradation intermediate from phenylalanine catabolism, was consistently upregulated from T2 through T4, suggesting active degradation of aromatic compounds, potentially including phthalates and PE-derived aromatics, through the phenylacetate degradation pathway [81]. According to Parshikov et al. [82], the marked downregulation of piperidine may reflect its consumption as a nitrogen source or its conversion to other metabolites through ring-opening reactions catalyzed by specialized bacterial enzymes. Integrated metabolic network analysis revealed strong interconnections between central carbon metabolism, lipid metabolism, and xenobiotic degradation pathways, suggesting coordinated regulation of these processes. This metabolic integration likely reflects the cellular necessity to balance energy generation, biosynthetic demands, and detoxification activities under xenobiotic stress.

### 4.3. Relevance for Soil Functioning and Agricultural Sustainability

The observed alterations in microbial community structure and metabolic pathway activity could have significant implications for soil ecosystem functioning and the long-term sustainability of PE mulch-based agricultural systems. The shift toward bacterial-dominated communities with concurrent fungal suppression represents fundamental soil food web restructuring that may compromise multiple ecosystem services. Suppression of saprotrophic fungi and mycorrhizal symbionts poses particular concerns for nutrient cycling and plant productivity. Saprotrophic fungi serve as primary decomposers of recalcitrant organic matter, particularly lignocellulosic plant residues, through the production of extracellular oxidative enzymes, including lignin peroxidases, manganese peroxidases, and laccases [63,71,72]. Decreased activity may reduce decomposition efficiency of crop residues and organic amendments, potentially altering carbon sequestration dynamics and soil organic matter quality [64]. Arbuscular mycorrhizal fungi abundance decrease is especially problematic for legume cultivation as these symbionts form extensive hyphal networks, increase phosphorus acquisition beyond the root depletion zone, and contribute to soil aggregate formation through glomalin production [75]. Like other legumes, field pea relies heavily on mycorrhizal associations for phosphorus nutrition, particularly in low-phosphorus soils [41]. Observed mycorrhizal suppression may therefore necessitate increased phosphorus fertilization to maintain crop productivity, potentially offsetting some of the resource-use efficiency benefits of mulching practices. Metabolic pathway perturbations indicate potential constraints on the soil nitrogen cycling capacity. The glutamic acid and N-α-acetyl-L-citrulline downregulation suggests reduced nitrogen assimilation and transformation activity, affecting nitrogen availability for plant uptake. This phenomenon is particularly relevant for legume systems, where soil nitrogen dynamics interact with biological nitrogen fixation through rhizobial symbiosis [53,61]. Enhanced anaerobic bacterial activity and associated fermentative metabolism may also influence nitrogen cycling through altered denitrification and anaerobic ammonium oxidation processes, potentially increasing gaseous nitrogen losses and reducing nitrogen use efficiency. The activation of xenobiotic degradation pathways demonstrates the adaptive capacity of microbes to PE-derived compounds; however, this metabolic investment may impose fitness costs. Xenobiotic metabolism typically requires specialized enzyme system induction and may divert cellular resources from growth and reproduction toward detoxification and stress tolerance [10,49]. The observed metabolic shifts toward maintenance metabolism—evidenced by reduced glycolytic flux, altered energy metabolism, and modified metabolite profiles [74]—suggest that microbial communities operate under chronic stress conditions imposed by PE mulch emissions.

## 5. Conclusions

This study provides the first integrated field-based evidence that polyethylene (PE) mulch emissions disrupt soil microbial communities and metabolic processes through the continuous release of polymer-derived compounds into agricultural soil throughout the growing season. PE-derived alkanes, phthalates, and additives accumulation creates dual pressures—microenvironmental optimization and xenobiotic stress—that restructure the soil microbiome. Our findings revealed a 2.5-fold bacterial abundance increase with 40% fungal suppression, confirming selective microbial restructuring under PE-derived chemical stress. Bacterial proliferation, particularly among anaerobic bacteria and actinomycetes, coupled with fungal suppression, critically shifts the decomposer community structure, transitioning soil decomposition from oxidative, fungal-mediated pathways toward fermentative, bacterially driven metabolism. This reorganization of the decomposer food web has direct consequences for lignocellulose decomposition efficiency, soil organic matter stabilization, and greenhouse gas dynamics. Metabolomic profiling revealed that PE emissions trigger coordinated metabolic reprogramming, characterized by increasing fermentative pathways, suppressing nitrogen assimilation, and activating the xenobiotic biodegradation pathways. The 3.2-fold upregulation of fermentation intermediates (lactate and acetate) combined with glutamic acid and N-α-acetyl-L-citrulline downregulation provides mechanistic evidence of impaired nitrogen cycling, directly linking PE emissions to reduced nitrogen use efficiency. Chronic stress-induced maintenance metabolism diverts cellular resources from growth to detoxification, imposing a sustained metabolic cost on the soil microbial community. The suppression of nitrogen metabolism intermediates and mycorrhizal fungi particularly threatens legume systems, compromising biological nitrogen fixation and phosphorus acquisition. This creates a previously uncharacterized paradox: PE mulching, applied to enhance crop productivity, systematically erodes the biological mechanisms that make legume cropping systems nitrogen-efficient and environmentally sustainable. While PE mulching provides agronomic benefits through microclimate regulation, the observed biochemical alterations demonstrate trade-offs in long-term soil health that extend beyond physical soil conditioning. Short-term productivity gains may be offset by long-term biological degradation, challenging the agricultural sustainability of plastic mulch use in intensive cropping systems. These findings advance current understanding by establishing a metabolic link between PE mulch emissions, soil microbiome restructuring, and metabolic pathway perturbation—a connection not previously demonstrated in an integrated field-based study. Future research should employ metagenomics and metatranscriptomics to characterize microbial responses under extended mulching regimes and to determine whether the observed perturbations are reversible following mulch removal. Developing alternative mulching materials with reduced chemical emissions is critical for sustainable agricultural intensification. This research provides a foundation for designing next-generation biodegradable mulches that maintain agronomic benefits while minimizing soil biochemical disruption.

## Figures and Tables

**Table 1:** Two-way ANOVA results and post hoc comparisons of polyethylene mulch-emitted organic compounds in field pea (*Pisum sativum* L.) cultivation soil.

Compounds	G		T		G × T		Post Hoc Comparisons PE vs. C				
	*F*_(1,40)_	*p*	*F*_(4,40)_	*p*	*F*_(4,40)_	*p*	T0	T1	T2	T3	T4
n-Undecane	94.85	***	18.01	***	12.90	***	***	***	***	***	***
n-Dodecane	808.96	***	57.42	***	55.29	***	***	***	***	***	***
2-Methyl-dodecane	10.44	**	0.45	ns	0.38	ns	*	ns	ns	ns	ns
n-Heptyl-cyclohexane	793.61	***	63.91	***	63.3	***	**	***	***	***	***
n-Tridecane	318.49	***	15.26	***	15.14	***	***	***	***	***	***
n-Tetradecane	215.94	***	11.6	***	11.47	***	ns	***	***	***	***
n-Pentadecane	345.83	***	17.83	***	17.92	***	***	***	***	***	***
n-Hexadecane	17.85	**	1.23	ns	1.24	ns	ns	ns	ns	ns	ns
n-Nonyl cyclohexane	379.86	***	13.88	***	13.47	***	***	***	***	***	***
Dimethyl phthalate	134.39	***	3.85	*	3.63	*	***	***	***	***	***
Di-n-octyl phthalate	32.78	***	1.47	ns	1.48	ns	ns	**	ns	*	ns
Diethyl phthalate	98.3	***	3.03	*	2.91	*	***	***	***	***	***
Dibutyl phthalate	29.72	***	2.28	ns	2.25	ns	ns	*	**	***	***
Benzyl-butyl phthalate	324.3	***	17.49	***	17.15	***	***	***	***	***	***
Bis-2-ethylhexyl-phthalate	147.96	***	4.3	**	4.2	**	***	***	***	***	***
Acetyl tributyl citrate	66.94	***	2.84	*	2.61	*	*	***	***	***	***
Bis-2-ethylhexyl adipate	97.47	***	3.33	*	3.2	*	**	***	***	***	***
Naphthalene	359.39	***	13.87	***	13.62	***	ns	***	***	***	***

* *p* < 0.5, ** *p* < 0.01, *** *p* < 0.001, and ns—nonsignificant.

**Table 2:** Two-way ANOVA results for the effects of polyethylene mulching and sampling time on the bacterial and fungal communities of the *Pisum sativum L*. cultivated soil.

Microbial Group	C (nmol·g^−1^)	PE (nmol·g^−1^)	G		T		G × T		Contrast Estimate C-PE		Direction
			*F*_(1,40)_	*p*	*F*_(4,40)_	*p*	*F*_(4,40)_	*p*	*F*_(1,40)_	*p*	
**Bacteria**											
Gram-negative	55.8 ± 9.8	61.6 ± 13.7	4.25	*	5.78	***	1.06	ns	5.88	*	PE > C
Gram-positive	20.3 ± 6.02	25.9 ± 8.4	15.03	***	12.84	***	1.93	ns	5.59	***	PE > C
Aerobic	23.4 ± 4.4	35.8 ± 10.5	74.83	***	14.43	***	5.79	***	12.4	***	PE > C
Anaerobic	12.1 ± 3.3	25.7 ± 9.7	143.88	***	19.84	***	9.36	***	13.57	***	PE > C
Actinomycetes	21.8 ± 4.4	34.7 ± 9.6	93.02	***	14.58	***	5.43	**	12.83	***	PE > C
**Fungi**											
Arbuscular Mycorrhizal	17.7 ± 4.3	12.5 ± 3.8	23.04	***	2.16	ns	1.54	ns	5.18	***	C > PE
Saprotrophic	6.0 ± 1.4	4.01 ± 1.4	26.59	***	0.93	ns	2.17	ns	1.96	***	C > PE
Ectomycorrhizal	3.7 ± 0.93	2.92 ± 0.84	17.29	***	8.84	***	0.66	ns	0.82	***	C > PE

* *p* < 0.5, ** *p* < 0.01, *** *p* < 0.001, and ns—nonsignificant.

## Data Availability

The original contributions presented in this study are included in the article. Further inquiries can be directed to the corresponding author.
